# Drug abusers have impaired cerebral oxygenation and cognition during exercise

**DOI:** 10.1371/journal.pone.0188030

**Published:** 2017-11-10

**Authors:** Kell Grandjean da Costa, Vanessa Soares Rachetti, Weslley Quirino Alves da Silva, Daniel Aranha Rego Cabral, Daniel Gomes da Silva Machado, Eduardo Caldas Costa, Rodrigo Menezes Forti, Rickson Coelho Mesquita, Hassan Mohamed Elsangedy, Alexandre Hideki Okano, Eduardo Bodnariuc Fontes

**Affiliations:** 1 Federal University of Rio Grande do Norte (UFRN), Department of Physical Education, NEUROEX–Research Group in Physical Activity, Cognition and Behavior, Natal, RN, Brazil; 2 Postgraduate Program in Health Sciences, Federal University of Rio Grande do Norte, Natal, RN, Brazil; 3 Federal University of Rio Grande do Norte (UFRN), Biophysics and Pharmacology Department, Natal, RN, Brazil; 4 Londrina State University, Center of Physical Education and Sports, Londrina, PR, Brazil; 5 Institute of Physics, University of Campinas, Campinas, SP, Brazil; 6 Brazilian Institute of Neuroscience and Neurotechnology, Campinas, SP, Brazil; 7 Center of Mathematics Computation and Cognition, Federal University of ABC, Santo André, SP, Brazil; Sao Paulo State University, BRAZIL

## Abstract

**Background:**

Individuals with Substance Use Disorder (SUD) have lower baseline metabolic activity of the prefrontal cortex (PFC) associated with impairment of cognitive functions in decision-making and inhibitory control. Aerobic exercise has shown to improve PFC function and cognitive performance, however, its effects on SUD individuals remain unclear.

**Purpose:**

To verify the cognitive performance and oxygenation of the PFC during an incremental exercise in SUD individuals.

**Methods:**

Fourteen individuals under SUD treatment performed a maximum graded exercise test on a cycle ergometer with continuous measurements of oxygen consumption, PFC oxygenation, and inhibitory control (Stroop test) every two minutes of exercise at different intensities. Fifteen non-SUD individuals performed the same protocol and were used as control group.

**Results:**

Exercise increased oxyhemoglobin (O_2_Hb) and total hemoglobin (tHb) by 9% and 7%, respectively. However, when compared to a non-SUD group, this increase was lower at high intensities (p<0.001), and the inhibitory cognitive control was lower at rest and during exercise (p<0.007). In addition, PFC hemodynamics during exercise was inversely correlated with inhibitory cognitive performance (reaction time) (r = -0.62, p = 0.001), and a lower craving perception for the specific abused substance (p = 0.0189) was reported immediately after exercise.

**Conclusion:**

Despite SUD individuals having their PFC cerebral oxygenation increased during exercise, they presented lower cognition and oxygenation when compared to controls, especially at elevated intensities. These results may reinforce the role of exercise as an adjuvant treatment to improve PFC function and cognitive control in individuals with SUD.

## Introduction

Addiction to chemical substances is a public health issue worldwide [1]. The Diagnostic and Statistical Manual of Mental Disorders of the American Psychiatric Association (DSM-V 2013) classifies drug addiction as Substance Use Disorders (SUD) and Addictive Disorders. This classification is determined when the individual meets two or more criteria regarding the use of the psychoactive substance (abstinence, tolerance, craving, repeated attempts to stop use, social, personal, physical or psychological problems related to use, etc.) [2]. Regarding the SUD treatment, conventional approaches includes psychotherapy (e.g. cognitive behavior therapy) and pharmacological therapy [3] provided by therapeutic settings (e.g. hospitals and specific clinics). Moreover, biological [4] and social models [5] of drug addiction in literature describes the features, consequences and causality related to drug abuse.

The biological model shows that reward and pleasure feelings related to drug use are associated with a large release of dopamine neurotransmitter by the mesolimbic system [6]. Chronic use of psychoactive substances promotes functional and structural changes in the circuits modulated by dopamine, especially in the prefrontal cortex (PFC), which decrease the metabolic activity of this region [7,8]. In addition, deterioration of other prosencephalic regions has also been reported [9,10], with decreased oxygenation [11] and a reduction in gray matter volume of the anterior cingulate, orbitofrontal and prefrontal cortex [12]. Impairment in these structures has been accompanied by reduced cognitive function [13]; i.e. decreased impulsivity control and emotional regulation, which may contribute to inappropriate decision-making and inhibitory control with further drug using and drug-seeking behavior [8]. Chronic damage in the PFC of individuals with SUD reduces the capacity to inhibit their desire for consumption and control over drug using and seeking behavior [14]. In this sense, SUD is currently considered a brain disease [4]. Therefore, alternative treatment approaches that have potential to induce beneficial neuroplasticity on individuals with SUD should be considered [4].

In addition to the conventional SUD treatment approaches, aerobic exercise can be an alternative treatment option for individuals with SUD, given that it has positive effects on craving [15], withdrawal episodes [16] and relapse [17]. Aerobic exercise has been evolutionarily linked to brain development [18]. An acute session of aerobic exercise improves cognitive performance [19], and an aerobic exercise training program positively affects the PFC neuroplasticity in non-SUD individuals [20]. It has been proposed that increased cerebral oxygenation during and after an exercise session may be a possible mechanism that explains improvements in cognitive performance [21]. Using the near-infrared spectroscopy (NIRS) during exercise, a meta-analysis has demonstrated that increases in cerebral hemodynamics, particularly in the PFC, occurs in an positive intensity-dependent manner [22]. This is very important given that increased blood flow, oxygen, and glucose supply for the neurons from the PFC can be decisive in the performance of cognitive tasks [19,21]. However, little is known about the effects of exercise on the brains of individuals with SUD.

Considering that individuals with SUD have chronic damage in the PFC due the use of psychoactive substances which decreases metabolic activity of this region [7,8], it is reasonable to think that these individuals could present blunted PFC oxygenation during exercise in an intensity-dependent manner, which in turn would impair their cognitive performance. Therefore, we tested the hypothesis that individuals with SUD have impaired PFC oxygenation during exercise in an intensity-dependent manner compared to healthy control individuals. Moreover, a secondary aim of this study was to determine the exercise intensity that provides greater PFC oxygenation in individuals with SUD. Considering that increased cerebral oxygenation during exercise may be a possible mechanism that explains improvements in cognitive performance [21] and PFC function [19], it seems important to determine the exercise intensity that increases PFC oxygenation at a greater magnitude in order to optimize the exercise prescription for this population.

## Methods

### Subjects

Twenty-nine individuals were recruited and then divided into two groups. The SUD group consisted of 14 patients hospitalized in a public psychiatric hospital with daily ambulatory control and bio-psychosocial therapies for the treatment of chemical dependence. All subjects scored higher than 27 points on the Alcohol, Smoking, and Substance Involvement Screening Test (ASSIST), and met the criteria for substance use disorder in the Diagnostic and Statistical Manual of Mental Disorders of the American Psychiatric Association (DSM-V, 2013). There were no exclusion criteria for a specific substance, where the preferred substances (alcohol, nicotine, marijuana, cocaine/crack, LSD, amphetamines, hypnotic sedatives, and ecstasy) were defined by the ASSIST. In the SUD group, 35.5% reported to be addicted to one substance, 43% reported to be addicted to two substances, and 21.5% to three substances. The control group (CG) was composed of 15 healthy individuals invited to participate in the study through social media and were matched by gender, body max index (BMI) and physical fitness (VO_2_ peak and blood pressure). All CG subjects had no substance use disorders classified by the DSM-V criteria. The following inclusion/exclusion criteria were established using information from medical records and interviews with both groups: Inclusion criteria: age between 18 and 45 years; fulfill the specific DSM-V criteria for each group; stable clinical condition; do not present severe withdrawal symptoms; be able to read and write in Portuguese. Next, exclusion criteria were: a condition of intoxication or severe symptoms of withdrawal due to any substance use; dependence on some psychoactive substance (CG); cardiovascular risk, impaired mental status [measured by the Mini Mental Status Examination (MMSE) questionnaire] or high blood pressure. Two subjects from the SUD group were excluded, one for not meeting the criteria for withdrawal symptoms, and another due to MMSE score below 24 points. All subjects were informed about the study, and signed a consent form. The study followed the standards of the Helsinki Declaration and was approved by the local ethics committee.

### Study design

This is an experimental cross-sectional study with manipulation of exercise intensity as an independent variable. Subjects were first screened with questionnaires [Physical Activity Readiness Questionnaire (PAR-Q), MMSE, ASSIST and DSM-V criteria], had mean arterial pressure (MAP) measured and were familiarized with the procedures and exercise protocol. At the second meeting after a minimum of 48 hours, the volunteers performed the exercise protocol consisting of a maximal exercise test on a cycle ergometer accompanied by measures of cerebral hemodynamics and ergospirometry. All procedures follow the American Heart Association guidelines for cardiopulmonary testing [23]. Ratings of perceived exertion (RPE) were measured by Borg 6–20 scale every two minutes [24]. The test was discontinued when subjects reached maximal voluntary exhaustion as determined by the Borg scale between 18 to 20, they were unable to maintain the determined cadence rate (<5 rpm) for more than five seconds, or presented signs of extreme fatigue. Also, the participants performed the cognitive Stroop test every two minutes during exercise after the RPE measurement. In addition, the craving scale for substance use was applied prior to and immediately after exercise. Cerebral hemodynamics and cognitive performance were analyzed in the first two minutes of exercise (beginning) and in the exercise intensity of ventilatory threshold (VT), respiratory compensation point (RCP) and peak of oxygen consumption (VO_2_ peak). In addition, 13 subjects were reassessed to test the reproducibility of the protocol. The coefficient of variation (CV) and Pearson's correlation (r) were calculated for two measurements of the variables VO_2_ peak (mL/kg/min) (CV = 2.39%; r = 0.94), maximum load (Watts) (CV = 1.91%; r = 0.97), total test time (min) (CV = 3.2%, r = 0.84) and reaction time (ms) on the Stroop test (CV = 5%; r = 0.66).

### Procedures

#### Mini-Mental Status Examination (MMSE)

A translated (to Portuguese) and validated version of the test was used, which had also been applied before to a similar population [25]. This exam consists of 11 items that evaluate 5 areas of cognitive function: orientation, attention, calculation, memory and language. The maximum score allowed is 30 points. All individuals with scores between 24 and 30 points were considered to be without cognitive impairment.

#### Physical Activity Readiness Questionnaire (PAR-Q)

This questionnaire is composed of eight questions that aim at detecting cardiovascular risk and is considered a minimum standard of evaluation before participation in physical activities [26]. All subjects were classified as having no cardiovascular risk.

#### Alcohol, Smoking, and Substance Involvement Screening Test (ASSIST)

This questionnaire, developed by the World Health Organization (WHO, 2002), assesses the risks or problems related to the use of alcohol, marijuana, cocaine/crack, LSD, sedatives, hallucinogens, heroin, Inhalants, opioids, and other drugs. The questionnaire consists of seven questions that include a score and classifies the individual as being without the need for treatment (<3 pts), needing a brief intervention (> 4 pts), or an immediate intervention (> 27 pts), according to abused substance, and for which there may be more than one. The questionnaire has been widely used in several countries [27], and it was used in this study to identify the drug of preference, since all volunteers were on a regimented treatment.

#### Craving scale

The Brief Cocaine Craving Questionnaire [28] was used, translated to Portuguese and adapted to be used according to the abused substance [29]. This instrument consists of ten questions on the "feelings" and "thoughts" about the act of "using the substance". The participant is asked to answer each question using a seven-point likert scale ranging from "totally agree" to "totally disagree". When the abused substance of the participant was different than cocaine/crack, it was requested that the questions were answered according to their substance (of abuse). The sum of the points from each question was used to classify the desire for using the substance, and presented as a mean for the SUD group.

#### Cardiopulmonary exercise testing

Ergospirometry was performed during the incremental maximal exercise test on a bicycle ergometer (model CG04, Inbrasport, Porto Alegre, Brazil). The initial intensity was 25 W with increments of 25 W every two minutes, and cadence was maintained between 60–70 r/min. Minute volume (VE), oxygen consumption (VO_2_) and carbon dioxide production (VCO_2_) were measured by a metabolic analyzer (Metalyzer® 3B). Threshold determinations and maximal oxygen consumption (VO_2_max) were performed using software (Ergo PC Elite®) supplied by the manufacturer. Ventilatory threshold (VT) was determined by the equivalent method when the VE/VO_2_ ratio presented its minimum value before presenting progressive increases. Respiration compensation point (RCP) was performed by the V-slope [30]. The equipment for gas exchange analysis was calibrated prior to each analysis. Calibration was performed with ambient gas samples (20.9% O_2_ and 0.04% CO_2_) and samples obtained from a cylinder with a known concentration of O_2_ (16%) and CO_2_ (5%). In addition, the gas flow of the apparatus was calibrated using a three-liter syringe. All procedures were performed according to the manufacturer's specifications.

#### Cerebral hemodynamics

Cerebral oxygenation was analyzed using Near Infrared Spectroscopy (NIRS) (Imagent, ISS, Champaign, IL, USA) which allows absolute quantification of oxyhemoglobin (O_2_Hb), deoxyhemoglobin (HHb) and total hemoglobin [(tHb) = O_2_Hb + HHb] according to absorbance at specific wavelengths using the modified Beer-Lambert law [31]. Each one of the two optodes contained one detector and four pairs of sources, with each pair consisting of two wavelengths (690 and 830 nm) and positioned at different distances from the detector (1.5, 2.5, 3.5, and 4.5 cm). According to the international EEG 10–20 distribution system, the optodes were placed on the participant’s PFC on the Fp1, Fpz and Fp2 regions. A dark bandage was tied to prevent any light entering the PFC. The equipment was calibrated before each evaluation using a calibration phantom and a check phantom that have specific absorption and scattering coefficients. Data were analyzed by a specific script in Matlab and Homer 2 [32], provided by the National Health Institutes. Data from the six minutes of rest were exported and normalized to express the magnitude of the changes (Δ), and thus we were able to analyze the exercise changes in the cortex. Results are presented and analyzed with data of the two optodes together.

#### Stroop test

A computerized version of the Stroop test was presented using Testinpacs®. The tasks during exercise were viewed on a computer screen at 90 cm distance in front of the volunteer. Responses for a set of tasks were given as fast as possible through two buttons attached to the bicycle handlebar. The cognitive portion of the Stroop Test has been used as a psychometric test to evaluate cognition, as it is related to the executive functions of decision-making and inhibitory control exercised by the PFC. The Stroop effect is based on task situations in which color names are written in different color fonts. The individual should inhibit the meaning of the word and choose the correct answer corresponding to the color in which the word is written [33]. The current study used a Stroop test that had three stages: The first two stages were control conditions (indicate the ink color of objects; indicate the color word that appears in white ink) and the last stage was incongruent (respond to the ink color of colored words). In the first stage, rectangles in green, blue, black and red colors were displayed in the center of the screen. At the same time, responses that matched or mismatched the color of the rectangle were displayed in the lower corners of the screen. Each stimulus was presented until the participant indicated the color in the lower corners corresponding to the color of the rectangle. In the second stage, the name of one of the aforementioned colors was displayed in white font. Lastly, on the third and final stage of the test, the name of one of the four colors was displayed with a different font color. Participants were instructed to press the button corresponding to the color of the font and ignore the name of the color. The stimuli were presented randomly at all stages, (12 trials per stage). The average time to complete the task was approximately 45s. The reaction time (RT) in milliseconds and the number of errors (n) committed in each stage were recorded. Only the RT of the correct responses and of response time longer than 200 ms were used for analysis. To analyze the data, we calculated the mean of the incongruent stage (12 trials) less the mean of the 2 congruent stages (24 trials), and only the error rate for the incongruent phase, which is a proxy for the inhibition of color word responses (inhibitory control). Average reaction time (ms) was compared between the groups at rest and among the exercise intensities, while mean errors at rest and exercise were compared together between groups.

#### Statistical analysis

Data are expressed as mean and standard deviation (SD). The Shapiro-Wilk test was used for data normality analysis. Unpaired Student’s t-test was used to compare subjects’ characteristics between groups as age, BMI, MAP, number of errors on the Stroop test, VO_2_ peak, maximum load, and total test time. Paired Student's t-test was used to compare the score on the fissure scale (pre x Post) in the SUD group. The Mann-Whitney test was used for the non-parametric data. A two-factor group (Control and SUD) x intensity (Beginning, VT, RCP and VO2 peak) mixed-model repeated measures ANOVA analysis was conducted to compare brain hemodynamics (tHb, O_2_Hb, HHb) and cognition (Time reaction). Whenever the sphericity assumption was violated, the degrees of freedom were adjusted and reported using the Greenhouse-Geisser epsilon correction. Partial eta squared (η^2^p) was used to determine the effect size of these analyses. If necessary, Bonferroni post hoc test was used to determine where the significant differences occurred. For correlation between cerebral oxygenation and cognitive performance, the Pearson correlation was used for parametric data and the Spearman correlation for non-parametric data. The level of significance was set at p <0.05. SPSS® 20.0 for Windows (SPSS, Inc., Chicago, IL) was used for statistical analysis and GraphPad Prism software was used for graphing.

## Results

### Groups characteristics

There was no difference (p>0.05) in the BMI, MAP, VO_2_ peak or the maximum load (Table 1) between groups. Additionally, the SUD group were older than the control group (U = 42, p = 0.0062).

**Table 1:** Characteristics of SUD and control groups.

	SUD Group (n = 14)	Control Group (n = 15)	p-value
Age (years)^#^	33 (20–49)	25 (18–32)*	0.0062
BMI (kg/m^2^)	27.9 ± 3.5	23.7 ± 3.3	0.2100
MAP(mmHg)	95.1 ± 7.8	92.1 ± 9.1	0.5824
VO_2_ peak (ml/min/kg)	24.6 ± 5.2	26.6 ± 4.8	0.3158
Maximum load (Watts)^#^	135 (85–185)	145 (105–250)	0.1996
Total test time (min)	16 ± 2.0	15 ± 3.0	0.5467

**Legend**: Age, body composition (BMI), and fitness (MAP (baseline), VO_2_ peak, Maximum load and total time reached during the incremental test) compared between the SUD group and control group.

**Note:** data expressed as median, mean and standard deviation; BMI = Body mass index; MAP = Mean arterial pressure.

*significant difference

# Median values with confidence interval (95% Cl).

The SUD group presented individuals with crack/cocaine, alcohol, and marijuana dependence; 42% of the sample was classified as exclusively using crack/cocaine, 21.4% alcohol and 14.2% marijuana. In addition, 64.2% of the SUD group were addicted to tobacco, 14.2% were addicted to Crack/Cocaine and alcohol together, and 7.2% to marijuana and alcohol. Additionally, 100% of the sample reported having used a least one, two or more other psychoactive substances (LSD, amphetamines, ecstasy, inhalants, hypnotics and sedatives). The 95% confidence interval (95% CI) for drug use ranged from 12 to 21 years, abstinence values ranged from 2 to 8 months, and MMSE score ranged from 26 to 29 points. These specific characteristics can be seen in Table 2 for each group of drug preference.

**Table 2:** Drug preferences of SUD group.

Drug Preference Groups (>27 points ASSIST)				
Abused Drugs	Time used (years)	Abstinence (months)	Tobacco use (%)	MMSE (points)
Crack/Cocaine (n = 8)	17 ± 4.2	5.5 ± 4.2	85.8	26 ± 1.6
Alcohol (n = 6)	16.5 ± 5.5	4.8 ± 3.4	66.6	27.3 ± 1.7
Marijuana (n = 3)	11.6 ± 5.7	3.3 ± 0.8	33.3	28.3 ± 1.5

**Legend:** Number of subjects with his specific drug addiction, based on the substance that scored more than 27 points on the ASSIST questionnaire. Specific characteristics (Time used, abstinence, Tobacco use and MMSE score) of each group are also included. MMSE = Mini-Mental Status Examination

### Exercise induced results in cognition

**Fig 1:**
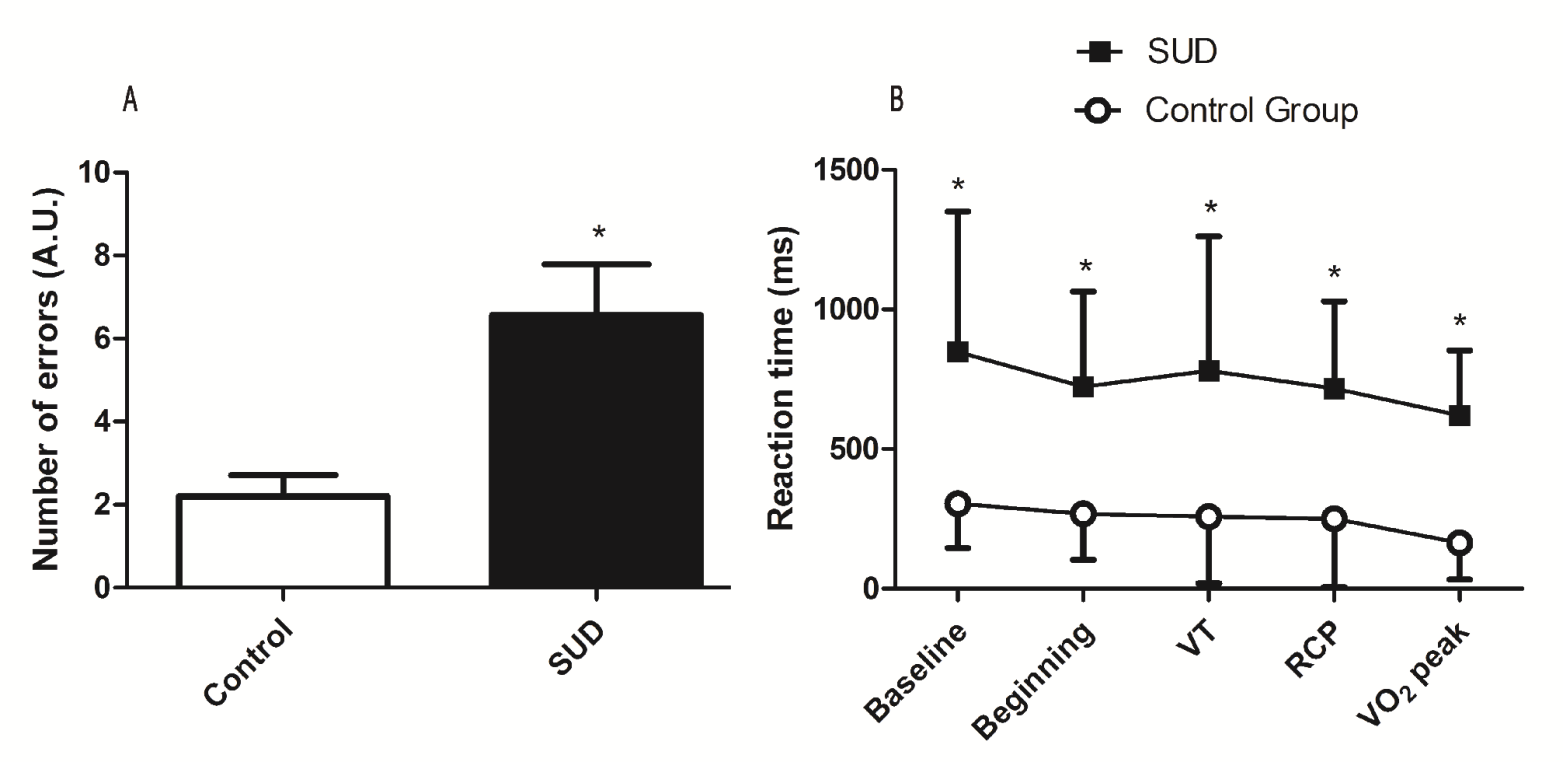
Cognitive performance at baseline and during exercise. Legend **A:** Mean errors during baseline and exercise. **B:** Reaction time before and during exercise; *(**A:** p = 0.0067, **B:** p<0.01).

Individuals with SUD have shown lower cognitive performance during rest and exercise. Fig 1 shows the number of errors and reaction time on the Stroop Test during rest and exercise protocol.

The SUD group presented an error rate of 11% compared to 4% of CG, and was significantly lower (U = 43, p = 0.0067) (Fig 1A). Fig 1B shows the reaction times during rest and exercise at different intensities. There was not a significant interaction for Stroop reaction time (F_(2.93, 75.98)_ = 2.387, p = 0.077) or main effect of intensity (F_(2.93, 74.98)_ = 0.759, p = 0.518). However, there was a significant group effect (F_(1, 26)_ = 16.78, p = 0.0001, η^2^p = 0.392). Bonferroni post hoc test revealed that reaction time was lower at rest and at all exercise intensities in the SUD group when compared to the CG (p<0.01).

### Exercise induced results in brain hemodynamics

**Fig 2:**
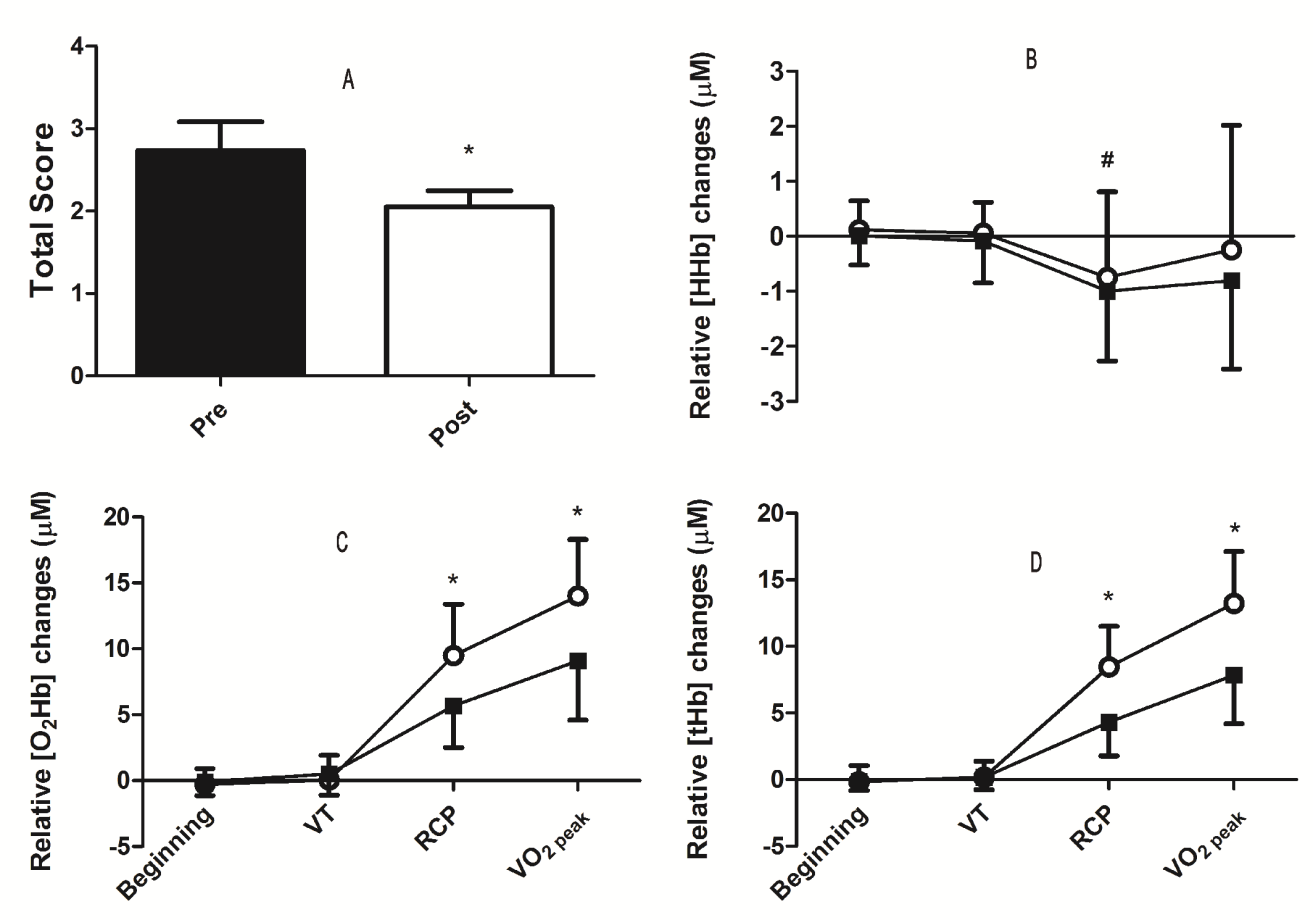
Exercise induced results in brain hemodynamics and craving. Legend: **A**: Craving Scale; **B**: Deoxyhemoglobin; **C**: Oxyhemoglobin **D:** Total hemoglobin; **VT**: Ventilatory threshold; **RCP**: Respiratory compensation point. *significantly different compared to control (**C**: p = 0.00005 and p = 0.00005; **D**: p = 0.00001 and p = 0.001).#significantly different compared to beginning (SUD; p = 0.001; CG; p = 0.004) and VT (SUD; p = 0.007; CG;p = 0.014).

The incremental test of exercise increased the PFC hemodynamics in both groups. However, it was lower in the SUD group at high intensities (RCP and VO_2_ peak). After the protocol, the SUD group also shown lower craving scores for the specific drug of abuse. Fig 2 shows acute effects of exercise on the craving scale (Pre x Post) and prefrontal cortex hemodynamics in the first 2 minutes (beginning) and at three relative intensities (VT, RCP, and VO_2_ peak) during the incremental maximum test in individuals with substance use disorder (SUD) compared to a control group.

SUD group presented a significant decrease (t_(14)_ = 2.64, p = 0.0189) on the craving scale after exercise (Pre 2.73 ± 1.36 vs. Post 2.05 ± 0.75). There was an exercise effect which increased O_2_Hb and tHb concentrations by 9% and 7% compared to baseline (Fig 2C and 2D). For O_2_Hb, there was a significant main intensity effect (F_(1.59, 87.95)_ = 15.09, p = 0.0001, η^2^p = 0.215), a significant group interaction by intensity (F _(1.59, 87.95)_ = 15.01, p = 0.0001, η^2^p = 0.214), and a main group effect (F_(1,55)_ = 16.67, p = 0.0001, η^2^p = 0.217). For tHb, there was a significant main intensity effect (F _(1.37, 75.83)_ = 9.09, p = 0.0001, η^2^p = 0.142), a significant group interaction by intensity (F_(1.37, 75.83)_ = 17.01, p = 0.0001, η^2^p = 0.236) and a significant group effect (F_(1,55)_ = 16.16, p = 0.0001, η^2^p = 0.227). Bonferroni post hoc test revealed that the concentrations of O_2_Hb and tHb at high intensities (RCP and VO_2_ peak) was higher compared to lower intensities (Beginning and VT) (p<0.001). Moreover, Bonferroni post hoc test also reveal that SUD group presented significantly lower concentrations (p<0.001) of O_2_Hb and tHb (Fig 2B and 2D) at high intensities. For HHb concentrations, RCP and VO_2_ peak intensities showed a decrease without a difference between groups (Fig 1B). Furthermore, there was a significant main intensity effect (F_(1.344, 75.346)_ = 10.741, p = 0.001, η^2^p = 0.161), no significant group interaction by intensity (F_(1.344, 75.246)_ = 0.5, p = 0.535) or main group effect (F_(1, 56)_ = 1.256, p = 0.267). Bonferroni post hoc test revealed that the concentrations of HHb was lower than VT at RCP intensity, and beginning intensities for both groups (p<0.05), without a difference between groups (p>0.5).

**Fig 3:**
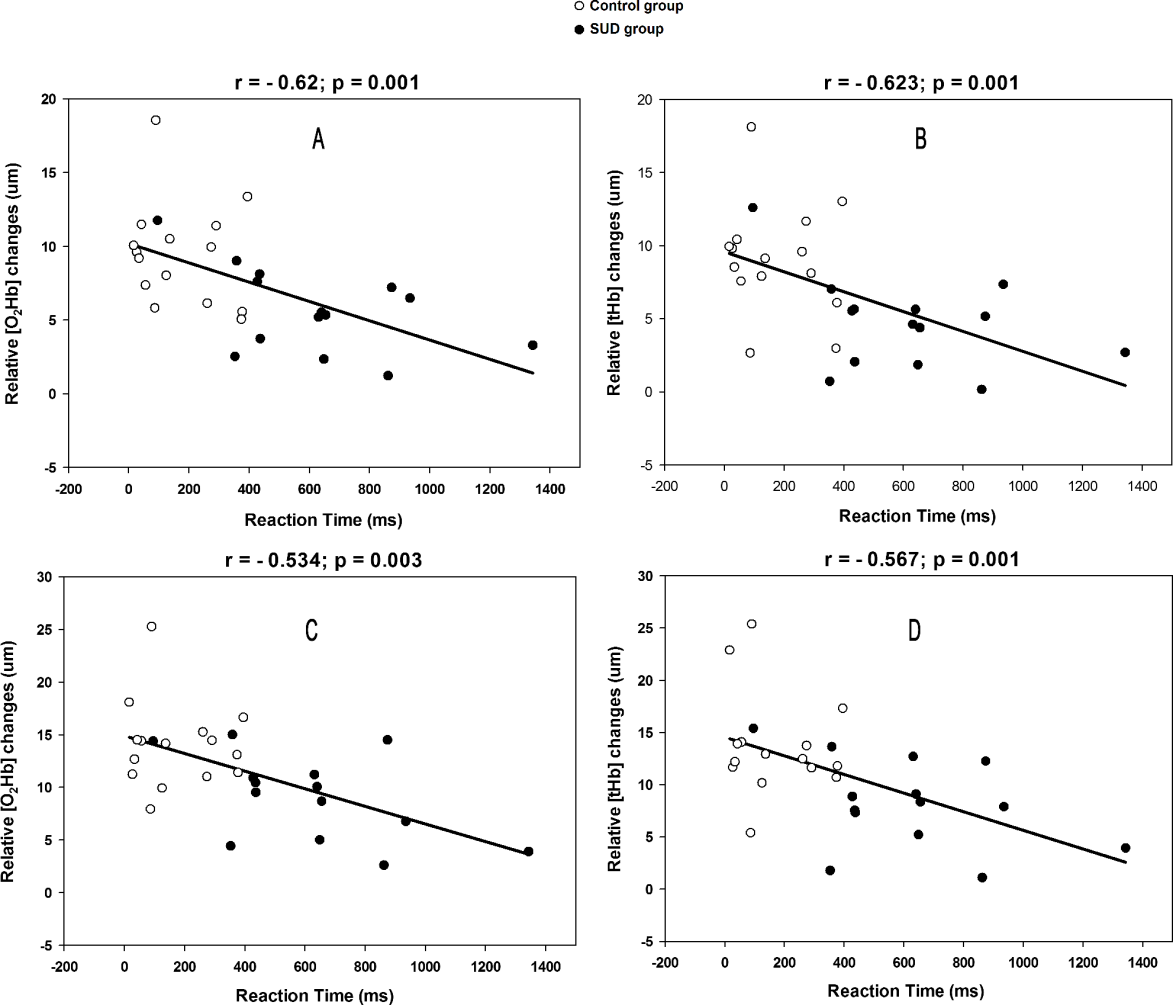
Exercise induced results in correlations. Legend: **A**: oxyhemoglobin (O_2_Hb) versus reaction time at RCP; **B**: total hemoglobin (tHb) versus reaction time at RCP; **C**: oxyhemoglobin (O_2_Hb) versus reaction time at VO_2_ peak; **D**: total hemoglobin (tHb) versus reaction time at VO_2_ peak.

Moreover, the increase of brain hemodynamics was associated with cognitive performance by a significant negative correlation (p<0.05) between PFC oxygenation (O_2_Hb) and blood volume (tHb) with cognitive performance (reaction Time) in the intensities of RCP and VO_2_ peak in both groups together, as shown in Fig 3. The black dots represents the SUD group and the white dots the control group. However, no significant association (p>0.05) was found when we split the groups for the correlations.

## Discussion

Our results show that higher exercise intensities promoted increased PFC oxygenation (O_2_Hb and HHb) and blood volume (tHb) in the SUD and control groups. However, these changes were lower in a population with a pathological condition of chemical dependence. These are the first results to show that the PFC function is impaired in individuals with SUD during exercise. In addition, patients with SUD also presented lower cognitive performance at rest and throughout the exercise. We also found a negative correlation between cerebral oxygenation and cognitive performance during exercise at elevated intensities (RCP and VO_2_ peak), and craving for the specific substance decreased after the exercise session.

Historically, it has been argued that cerebral blood flow (CBF) remains constant in different situations [34]. However, recent studies have shown changes in CBF during exercise [22] and even during sleep [35]. Exercise has been shown to promote an increase in CBF in response to physiological, metabolic and neuronal changes [36]. Despite the ability of exercise to modulate cerebral hemodynamics, changes in oxygenation have been associated with a slight decrease in HHb and an increase in O_2_Hb, while tHb represents the blood volume [37]. In the present study, we showed that O_2_Hb and tHb increased and HHb decreased (Fig 2). We believe that these results may indicate that both groups increased oxygen supply to the PFC during exercise, in particular at high intensities (RCP and VO_2_ peak). It is largely accepted that cognitive tasks increase brain metabolic activity with an increase of blood flow to provide glucose and oxygen to the active neurons [19]. Moreover, this augmented oxygenation in the PFC during incremental exercise has also been associated with physiological and cognitive demand while interpreting afferent signaling from cardiovascular and working muscles [38]. Thus, even with impaired PFC function due to substance abuse, the SUD patients were also able to increase cerebral oxygenation through exercise.

In studies with functional magnetic resonance imaging (fMRI), patients with alcohol, cocaine, and methamphetamine dependence have demonstrated lower PFC function in resting state situations [4,8]. This appears to be associated with a lower concentration of dopamine receptors (D2) in areas of the PFC, and a lower firing rate of dopaminergic neurons that project their axons from the ventral tegmental area to areas of the reward system (i.e. PFC, nucleus accumbens, striatum, hippocampus, and amygdala) [39]. Moreover, the decrease in metabolism in this region may also be associated with decreased blood flow caused by lower vascularization [8]. Our study is the first to demonstrate that PFC oxygenation is also impaired in situations of elevated body metabolism, such as exercising at high intensities. Furthermore, it has been shown that higher physical fitness is associated with improved cognition [20], which may be due to the release of molecules during exercise such as BDNF (Brain-derived neurotrophic factor) and VEGF (Vascular Endothelial Growth Factor) which promote structural changes in the PFC [40]. It is important to highlight that both groups (control and SUD) were paired by gender, BMI and physical fitness (Table 1), which may indicate that the lower oxygenation and cognitive performance in the SUD group might be due to structural and functional depletion of the PFC caused by the chronic use of chemical substances.

Decreased PFC oxygenation may induce impairments in important executive functions for drug abuse rehabilitation, such as decision-making and inhibitory control [14]. Decision-making is a process of choosing the best possible option out of the many alternatives, valuing future consequences that are more advantageous or avoiding disadvantageous consequences [41]. Also, inhibitory control is related to the ability to inhibit competitive, unpleasant or negative responses [14]. With these functions impaired in individuals with SUD, choices are made to obtain the immediate reward, promoting the relief of undesirable mental states, even with future disadvantageous consequences [14]. The loss of cognitive control over the desire for substance use has been related to the lower cerebral function of the PFC [42]. Our data is in agreement with these assumptions since the SUD patients had lower cognitive performance in the Stroop test at rest and throughout exercise when compared to the control group (Fig 1). Thus, these results may reinforce the decrements in cognition due to long-term drug abuse.

During exercise, the PFC has been suggested to play a key role in cognitive control and tolerance to physical exercise [43]. With PFC functions impaired, it is possible to believe that these individuals would have lower tolerance to physical activity. In addition, physical fitness has been proposed to be influenced by the ability of individuals to overcome the negative affective states produced by homeostasis changes promoted by exercise [44]. According to the somatic marker hypothesis, these adverse feelings are produced by changes in body states (emotions), and decision-making is a cognitive top-down process performed by the PFC based on the afferent information (bottom-up) from bodily states [45]. Thus, PFC dysfunction has also been related to impaired cognitive control to alleviate situations of negative affective states, mainly due to decreased top-down control over responses of bodily states sent to the PFC by the amygdala [46]. This dysfunction has also been demonstrated in individuals with SUD for their inability to inhibit the craving emotions for substance use produced by brain subcortical structures [47]. Therefore, the lower PFC oxygenation at high intensity and lower cognition in patients with SUD found in the present study may indicate caution when prescribing physical activity with elevated metabolic demand for individuals with SUD. Impaired executive functions of decision-making and inhibitory control may result in higher intolerance to homeostasis changes during exercise, leading to increased discomfort, and maybe lower adherence to exercise related to therapeutic rehabilitation.

Nevertheless, corroborating with other studies [15,16], we found that the individuals of the SUD group presented lower desire for substance abuse after the exercise protocol (Fig 2A). It has been demonstrated that acute physical exercise promotes increased dopamine concentration in regions of the reward system [48]. One of the explanations for this lower desire for the substance has been associated with the reward effects promoted after physical activity [49]. In addition, the increase in PFC oxygenation in these individuals promoted by exercise compared to rest may have contributed to better cognitive control over the desire to consume the drug [42], since the supply of oxygen and glucose with increased oxygenation and CBF during an incremental exercise seems to be decisive for the improvement of cognition after exercise [19]. In the same direction, a study showed improvement in the inhibitory control of methamphetamine-dependent individuals after an acute aerobic exercise session was associated with greater activity on electrophysiological records of the PFC [50]. Despite not having similar electrophysiological parameters to present here, it has been reported that the lower availability of O_2_Hb correlates with lower cognitive performance [51]. In this sense, our results (Fig 3) demonstrate an association of oxygenation and blood volume of the PFC with cognitive performance at higher intensity metabolic markers (i.e. RCP and VO_2_ peak). These results may also indicate the potential of oxygen availability (O_2_Hb) and blood flow (tHb) to improve cognitive performance, in which we postulate to be associated with increased control under the desire to use the substance after exercise.

Increased cerebral blood flow has been shown to be decisive in delivering nutrients (Glucose and O_2_) to the task-related regions [19,21]. However, it is argued that increased blood flow versus oxygen utilization obeys an exponential and non-linear relationship due to the uncoupling between glucose utilization and oxygen consumption, where a large increase in blood flow is necessary to increase neuronal metabolism in the region [31]. Moreover, the metabolic stress promoted by exercise and increased cerebral blood flow are associated with the activation of molecules BDNF and IGF-1 (insulin-like growth factor) that act together in signal transduction pathways by activating genes that increase synaptic activity [52]. In addition, gene activation by VEGF promotes angiogenesis to increase the supply of oxygen and glucose to the metabolism of these neurons [53]. Accordingly, our data showed an increase in oxygenation in the PFC that may contribute to increased metabolic activity in this region. We speculate that several acute effects of exercise accumulating in exercise session stimuli can promote chronic adaptations by altering neuroplasticity in the PFC, and consequently, improve cognition. These benefits may contribute to cognitive control over the drug using and seeking behavior in individuals with SUD. However, long-term studies verifying the effects of exercise on neurobiological and drug behavior markers are lacking in the literature.

## Limitations

Given the different functions and areas of the PFC (i.e. dorsolateral, ventromedial, orbitofrontal, anterior cingulate), one of our limitations is to measure general oxygenation of the PFC, and the NIRS technique does not reach the deep areas of the cerebral cortex. Despite this, the use of NIRS to evaluate brain function during exercise has been indicated due to less interference of movement artifacts [37]. Another possible limitation that can be discussed is the age difference between the evaluated groups, since the higher age in the SUD individuals could lead to cognition impairment; however, this may be more exacerbated in the elderly population [20] and declines in cognition in a low number of years (~8 years) need to be further demonstrated, particularly in adults. Moreover, there are no studies showing that middle-aged individuals are cognitively or have different brain structure from young adults. In this sense, age was a covariate during the ANOVA analysis, showing no main group effect for reaction time on the Stroop test (F _(1, 26)_ = 0.003, p = 0.937) or brain oxygenation (O_2_Hb) (F_(1,55)_ = 0.194, p = 0.66) and blood volume (tHb) (F_(1,55)_ = 0.127, p = 0.72). In addition, heterogeneity of drug use (cocaine/crack, alcohol, and marijuana) in the SUD group may be another limitation to be discussed because of the different mechanisms of drug actions in the neuronal synapses [39]. However, it is worth mentioning that there were individuals in the SUD group who presented two drugs of preference, and all the subjects had tried other types of psychoactive substances. Therefore, we believe that this heterogeneous sample characteristically represents this population. Furthermore, it was not possible to discontinue the medicinal treatment proposed by the hospital, where six volunteer patients used benzodiazepines which act on GABAergic receptors, hyperpolarizing neurons and reducing their firing rate. Consequently, this may have influenced the results of lower cognitive performance and oxygenation in the PFC presented in this study. However, this is a reality of the public psychiatric hospital that health professionals should be aware of, especially those who intend to use physical activity as a treatment option, even when it has proven many health benefits, but studies that evaluate the effects of physical exercise on neurobiological markers in this population are still lacking.

## Conclusion

Therefore, our results indicate that impairment of lower PFC function from chemical dependence can be transposed to high-intensity physical exercise situations. Such depleted function was related to a lower cognitive performance throughout the physical exercise. In addition, performing exercise at high intensity has shown to increase cerebral oxygenation of areas affected by the chronic use of substances. The possibility of altering the cerebral hemodynamics of chemical dependents through physical exercise strengthens its use as an alternative, non-invasive and non-pharmacological tool for the treatment of these patients. However, attention is necessary when working with high-intensity exercises in this population due to the lower PFC functioning. Impaired cognitive control during exercise may impair adherence and chronic benefits of physical activity. Moreover, new studies with this population in different treatment and drug addiction conditions are necessary to confirm our outcomes.

## Supporting information

S1 Dataset Data. (XLSX)
